# MicroRNA-218 inhibits the proliferation and metastasis of esophageal squamous cell carcinoma cells by targeting *BMI1*

**DOI:** 10.3892/ijmm.2015.2216

**Published:** 2015-05-21

**Authors:** TING WANG, TENGFEI CHEN, HUA NIU, CHANG LI, CHUN XU, YUANYUAN LI, RUI HUANG, JUN ZHAO, SHUYAN WU

**Affiliations:** 1 Department of Microbiology, Medical College of Soochow University, Suzhou, Jiangsu 215123, P.R. China; 2 Department of Thoracic and Cardiovascular Surgery, The First Affiliated Hospital of Soochow University, Medical College of Soochow University, Suzhou, Jiangsu 215006, P.R. China

**Keywords:** microRNA-218, esophageal squamous cell carcinoma, *BMI1*, proliferation, metastasis

## Abstract

MicroRNAs (miRNAs or miRs) play a pivotal role in esophageal carcinogenesis either as oncogenes or as tumor suppressor genes. In the present study, we found that the expression level of miR-218 was significantly reduced in esophageal squamous cell carcinoma (ESCC) tissues and ESCC cell lines. Moreover, its expression was found to correlate with the clinicopathological stage of ESCC; miR-218 expression was lower in the stage III tissue samples than in the stage I and II tissue samples. Furthermore, the decreased expression of miR-218 was found to be associated with an enhanced ESCC cell proliferation and metastasis. Western blot analysis and luciferase reporter assay revealed that miR-218 decreased *BMI1* expression by binding to the putative binding sites in its 3′-untranslated region (3′-UTR). The *BMI1* mRNA expression levels were markedly increased and negatively correlated with the miR-218 expression level in the ESCC tissues. Functional analyses revealed that the restoration of miR-218 expression inhibited ESCC cell proliferation, migration and invasion and promoted apoptosis. The knockdown of *BMI1* by siRNA showed the same phenocopy as the effect of miR-218 on ESCC cells, indicating that *BMI1* was a major target of miR-218. In the present study, our findings confirm miR-218 as a tumor suppressor and identify *BMI1* as a novel target of miR-218 in ESCC. Therefore, miR-218 may prove to be a useful biomarker for monitoring the initiation and development of ESCC, and may thus be an effective therapeutic target in ESCC.

## Introduction

Esophageal cancer (EC) is one of the most common human malignancies with a high mortality rate worldwide, and is much more common (by 3–4-fold) in males than females (1). The etiological factors of EC have been well-established by epidemiological studies, investigating living conditions, diet, nutrition and genetic susceptibility. EC occurs in the middle or upper one-third of the esophagus in form of esophageal squamous cell carcinoma (ESCC), while in the lower one-third or junction of the esophagus and stomach, EC occurs as esophageal adenocarcinoma (EAC) (2). Only a small number of patients with EC survive for >5 years with surgical treatment, and >60% of patients succumb to the disease due to distant metastasis and local recurrence. One reason for the high mortality rate associated with EC is that the majority of patients are diagnosed at the advanced stage of EC when they hospitalized (3). Therefore, it is important to identify the biological markers of EC in order to improve the rate of early diagnosis and to develop new treatment strategies to combat the disease.

MicroRNAs (miRNAs or miRs) are a recently discovered class of short non-coding RNAs, approximately 18–24 nucleotides in length (4), which bind to the 3′-untranslated regions (3′-UTRs) of target mRNAs mainly through complementary base pairing in their mature form, leading to target mRNA degradation or the inhibition of protein synthesis, consequently regulating gene expression at the post-transcriptional level (5,6). miRNAs can serve as either oncogenes or tumor suppressor and play a vital role in cancer development and cellular processes, including proliferation, apoptosis and migration. Furthermore, it has been demonstrated that the abnormal expression of miRNAs is associated with the development and progression of cancer and has prognostic significance for ESCC (7,8). Therefore, miRNAs may be new biological markers for ESCC.

Previous studies have reported that miR-218 is significantly downregulated in ESCC tissues compared with adjacent non-cancerous tissues (9,10). In addition, recent studies have indicated that miR-218 inhibits cancer occurrence and development in various types of cancer, mainly through the inhibition of cancer cell proliferation and invasion by targeting cancer genes (11,12). However, the role of miR-218 in regulating ESCC development is poorly understood. *BMI1*, a component of the polycomb repressive complex 1, was initially identified as an oncogene that cooperates with c-Myc in inducing lymphomas in double transgenic mice (13). In the present study, we demonstrated the decreased expression of miR-218 and the high expression of *BMI1* in ESCC tissues from 33 clinical patients and in ESCC cell lines. We systematically verified that miR-218 targets *BMI1* and downregulates its expression in ESCC cells, and identified an inverse correlation between *BMI1* levels and miR-218 in ESCC cell lines and tissues.

## Materials and methods

### Patient sample collection

A total of 33 pairs of eligible esophageal mucosa samples from patients with ESCC were collected from the First Affiliated Hospital of Soochow University, Suzhou, China between July 2011 and April 2013. Each patient provided written informed consent for their tissue samples to be used for research purposes. The present study was approved by the Ethics Committee of Soochow University and the Scientific Advisory Panel of our institute.

### Cell culture

Human esophageal epithelial cells (HEECs) and ESCC cell lines (EC109, TE-1, EC9706 and KYSE150) were obtained from the Chinese Academy of Sciences (Shanghai, China) and cultured in RPMI-1640 medium (Invitrogen, Carlsbad, CA, USA) containing 10% fetal bovine serum (FBS) and 1% penicillin/streptomycin (Invitrogen). All cells were cultured in a humidified chamber containing 5% CO_2_ at 37°C.

### Bioinformatics analysis

TargetScan software (http://www.targetscan.org), miDRB software (http://mirdb.org/cgi-bin/search.cgi) and miRecords software (http://www.mirbase.org) use two ways to search for predicted miRNA targets. One is searching the name of the miRNA (enter the name of the required miRNA and view its predicted targets). Another is searching by gene target information (enter the GenBank Accession No., NCBI Gene ID or Gene Symbol and view the miRNAs which target the gene of interest.

### Construction of plasmids, cell transfection and dual-luciferase assay

To construct a *BMI1* overexpression plasmid, the *BMI1* expression construct was generated by PCR to amplify a 2293-bp fragment encoding the *BMI1* cDNA (without 3′-UTR) which was obtained by reverse transcription-polymerase chain reaction (RT-PCR) using RNA from the EC109 cells. The sense primer (5′-CGCGGATCCATGAGAGGCAGAGATCGGGG-3′) contains a *Bam*HI restriction site and the antisense primer (5′-CCGGAATTCAGGTCGAACCAGTTGGGAGA-3′) contains an *Eco*RI restriction site. The PCR products were purified and then cloned into pcDNA3.1(+) (Invitrogen). The construct was verified by DNA sequencing.

To construct a luciferase reporter plasmid containing the *BMI1* 3′-UTR fused to the 3′ end of a luciferase reporter gene, the psiCHECK-2 dual luciferase vector (Promega, Madison, WI, USA) was used. Briefly, a 388-bp fragment containing 2 predicted miR-218 target sites (position 1470–1477 and position 1751–1758) was amplified by PCR using the following primers: forward, 5′-CCGCTCGAGTGTTCATCACCCATCAGTTATT-3′ (underlined letters indicate the *Xho*I site) and reverse, 5′-ATAAGAATGCGGCCGCAGCAATGTATTCTCTTTAACG-3′ (underlined letters indicate the *Not*I site). The *Xho*I and *Not*I-digested PCR product was then cloned into a psiCHECK-2 vector digested with *Xho*I and *Not*I to generate the psiCHECK-2-*BMI1*-3′-UTR-wild type. To prepare mutants, 4 bases in the predicted miR-218 target sites were changed (Fig. 3D). The mutated fragment was directly synthesized (Genewiz, Beijing, China), digested with *Xho*I and *Not*I, and cloned into the psiCHECK-2 vector to generate the psiCHECK-2-*BMI1*-3′-UTR-mutant. Subsequently, the EC109 cells were plated in a 24-well plate and co-transfected with 50 ng of psiCHECK-2-*BMI1*-3′-UTR-wild type or psiCHECK-2-*BMI1*-3′-UTR-mutant type and with 20 nM of either miR-218 mimics (5′-UUGUGCUUGAUCUAACCAUGU-3′) or the miR negative control (miR-NC). A scrambled sequence (5′-UUCUCCGAACGUGUCACGUTT-3′) was used as the miR-NC. At 48 h post-transfection, the EC109 were collected, and the luciferase activities were measured using the Dual-Luciferase Reporter assay kit (Promega) on a TD-20/20 Luminometer (Turner Designs, Sunnyvale, CA, USA).

Each experiment was carried out in triplicate. The results were expressed as relative *Renilla* luciferase activities, which were obtained by normalization to Firefly luciferase activities. All the transient transfections, including transfection with anti-miR-218 (5′-ACAU GGUUAGAUCAAGCACAA-3′) and anti-miR-NC, were performed using Lipofectamine 2000 (Life Technologies, Carlsbad, CA, USA). The scrambled sequence (5′-CAGUACUUUUGUGUAGUACAA-3′) was used as the anti-miR-NC. The knockdown of BMI1 was performed by using BMI-siRNA (CAAGCAGAAAUGCAUCGAATT) (Genepharma, Shanghai, China). A scrambled sequence (5′-UUCUCCGAACGUGUCACGUTT-3′) was used as the control.

### RNA extraction and reverse transcription-quantitative PCR

Total RNA was extracted from the ESCC tissue samples and adjacent non-tumor tissues using TRIzol reagent (Invitrogen, Oslo, Norway) according to the manufacturer’s instructions. The amount of RNA was measured on a NanoDrop spectrophotometer (Thermo Fisher Scientific, Waltham, MA, USA). The synthesis of cDNA with reverse transcriptase (RT) was performed using a M-MLV First Strand kit (Life Technologies). The concept of a stem-loop RT primer was used to design the RT primer for mature miR-218. The primer sequences for miR-218 and U6 detection are listed in Table I. To analyze the expression of miRNA, quantitative PCR (qPCR) was performed using the Platinum SYBR-Green qPCR SuperMix-UDG (Invitrogen) and an ABI 7500 Real-Time PCR system (Applied Biosystems, Foster City, CA, USA). GAPDH mRNA and U6 small nuclear RNA (U6 snRNA) were used as endogenous controls to normalize *BMI1* and miR-218 input. Relative expression was calculated using the ΔΔC_t_ method.

### Western blot analysis

Total protein was extracted using RIPA buffer (Cell Signaling Technology, Danvers, MA, USA) containing protease inhibitor and phosphatase inhibitor cocktail (Sigma-Aldrich, St. Louis, MO, USA) at 72 h post-transfection and the protein concentration was measured using the BCA Protein Assay kit (Beyotime, Haimen, China). Protein (50 *µ*g) was separated on SDS-PAGE and transferred onto PVDF membranes. The membranes were blocked by 1% BSA for 1 h and incubated overnight at 4°C with the primary antibody (anti-BMI1, #5856S; Cell Signaling Technology; and anti-GAPDH, AP0063; Bioworld Technology, St. Louis Park, MN, USA). The membranes were washed 4 times for 15 min with TBST (Tris-buffered saline and Tween-20). The membranes were then incubated with anti-rabbit secondary antibodies (goat anti-rabbit, sc-2004; Santa Cruz Biotechnology, Santa Cruz, CA, USA) at room temperature for 2 h and washed again. Finally, the proteins were visualized using an ECL detection system (Pierce, Rockford, IL, USA). The band density was quantified using Quantity One 4.6 software (Bio-Rad Laboratories, Hercules, CA, USA).

### Colony formation assay

A total of 400 EC109 cells were seeded in a dish with a diameter of 6 cm and transfected with miR-218 mimics, anti-miR-218 or the negative control(NC), using Lipofectamine 2000 (Life Technologies). The cells were collected and seeded in a 6-well plate in triplicate at 24 h post-transfection. FBS then (0.2 ml) was added to each well after 5 days. Following incubation for another 9–10 days, the plates were gently washed with PBS. Colonies were visualized by staining with 0.1% crystal violet and the colony number was counted (>50 cells).

### Cell proliferation assay

Cells at the logarithmic phase were seeded into 96-well plates at a density of 4×10^4^ cells/well. Cell proliferation was determined by MTT assay and the 5-ethynyl-2′-deoxyuridine (EdU) (Cell Light EdU DNA imaging kit; Guangzhou RiboBio Co., Ltd., Guangzhou, China). MTT assay was used to evaluate cell proliferation using the CCK-8 kit (Beyotime, Haimen, China) to calculate the cell absorbance value. The optical density (OD) was measured daily over 4 consecutive days at a wavelength of 450 nm (OD450) to estimate the viable cell numbers.

Another method for the determination of cell proliferation was the labeling of the cells undergoing DNA replication with EdU. Cell proliferation was quantified by measurement of the uptake of EdU. At 48 h after transfection, EdU was discerned in the DNA synthesis (S phase) of the cell cycle during 2 h of incubation, and medium containing EdU and 4% paraformaldehyde was then added to fix cells at room temperature for 30 min. The fixed cells were incubated with glycine (2 mg/ml) for 5 min in a shaker, and washed with PBS for 5 min. Following permeabilzation with 0.5% Trion X-100 for 10 min, the cells were washed with PBS for 5 min, followed by the addition of 1X Apollo dyeing reaction buffer to each well for 30 min while protecting the cells from light in a shaker at room temperature. DNA was stained with 1X Hoechst 33342 for 30 min and washed with PBS 3 times. The nucleated cells incorporated with EdU were observed by under a fluorescence microscope (Olympus IX71; Olympus, Tokyo, Japan) and the proportion of EdU uptake was determined.

### Cell migration and Transwell invasion assay

A wound healing assay was used to assess cell migration. The transfected cells were plated in 6-well culture plates to form cell monolayer (approximately 80% confluence). Following serum starvation for 6 h, a wound was made using a sterile 200 *µ*l pipette tip to scrape off the cells followed by washing twice with PBS to remove the detached cells. The cells were then incubated with 1% FBS. Cell migration was monitored at 0 and 48 h after the wound was made. The distances between the migration fronts were measured using ImageJ software (National Institutes of Health, Bethesda, MD, USA). The assessment of the migration rate by the percentage wound closure was performed as previously described (14).

For invasion assay, a Transwell chamber (Corning Costar, Cambridge, MA, USA) coated with Matrigel was used (BD Biosciences, Franklin Lakes, NJ, USA). Cells in serum-free medium (200 *µ*l) were seeded into the top chamber of wells in a 24-Transwell plate with inserts of 8 *µ*m pores (Corning Costar) at a density of 2.5×10^5^ cells/ml, while the bottom chamber was filled with 500 *µ*l medium containing 20% FBS. Following incubation at 37°C in a humidified incubator with 5% CO_2_ for 24 h, the cells which had invaded to the bottom chamber were fixed with 4% paraformaldehyde at room temperature for 30 min, stained with 0.1% crystal violet for 15 min and the cell number was counted in 5 randomly selected fields under an inverted microscope (Olympus CKX41; Olympus).

### Apoptosis assay

The miR-218-transfected cells were harvested at 48 h post-transfection and the cell density was adjusted to 1×10^6^ cells/ml. Apoptosis was induced by the addition of 200 *µ*M hydrogen peroxide (H_2_O_2_) to each well for 30 min. The cells were stained using the Annexin V-FITC Apoptosis detection kit (Beyotime, Beijing, China) according to the manufacturer’s instructions. Briefly, the cells were harvested and washed twice with cold PBS, and then resuspended in 195 *µ*l binding buffer. Annexin V-FITC (5 *µ*l) was added to this suspension, followed by incubation for 10 min at room temperature in the dark. After washing and resuspension, the cells were centrifuged at 1000 rpm to pellet the cells. Finally, apoptosis was assessed by flow cytometry.

### Statistical analysis

All the results were performed at least 3 times and the data are presented as the means ± standard deviation (SD). The data were analyzed using the SPSS 17.0 software package and GraphPad Prism 5.02 software (GraphPad Software, San Diego, CA, USA). For the cell lines, differences between 2 groups were assessed by an unpaired t-test (two-tailed). Comparisons between the clinicopathological characteristics and the mRNA expression levels in the ESCC samples were performed using non-parametric tests (Mann-Whitney U test for 2 groups). The correlation between the expression of miR-218 and BMI1 mRNA was evaluated by calculation of Spearman’s correlation co-efficient. A value of P<0.05 was considered to indicate a statistically significant difference.

## Results

### Expression of miR-218 is decreased in ESCC tissues and cell lines

**Figure 1:**
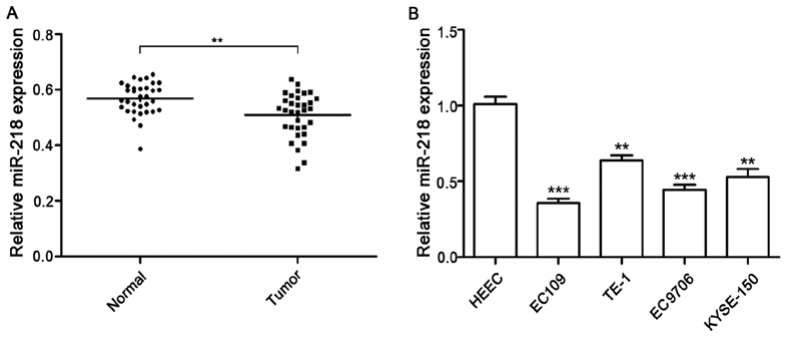
The decreased expression of miR-218 in esophageal squamous cell carcinoma (ESCC) tissues and ESCC cell lines. (A) Expression of miR-218 in 33 ESCC tissue samples and paired adjacent normal tissue samples detected by RT-qPCR. (B) Expression of miR-218 in human esophageal epithelial cells (HEECs) and 4 ESCC cell lines quantified by RT-qPCR. Data are presented as the means ± SD from 3 replicate samples. ^**^P<0.01, ^***^P<0.001.

In order to determine the role of miR-218 in ESCC, the miR-218 expression levels were determined in tissue samples from 33 patient with ESCC and their paired adjacent normal tissue samples. As shown in Fig. 1A, miR-218 expression was significantly decreased in the ESCC tissues when compared with the paired normal tissues. More importantly, the decreased expression of miR-218 was also associated with the clinicopathological stages of ESCC; the miR-218 expression levels were downregulated to a greater extent in the tissue samples from patients with stage III ESCC than those from patients with stageI and II ESCC (Table II). In addition, the decreased miR-218 expression correlated with the ESCC differentiation stage.

Moreover, the relative expression levels of miR-218 were measured in 5 cell lines. Compared with the HEECs, the miR-218 expression levels were markedly lower in the ESCC cell lines, including the EC109, TE-1, EC9706 and KYSE150 cells (Fig. 1B).

### miR-218 inhibits EC109 cell proliferation, migration and invasion, and induces apoptosis

**Figure 2:**
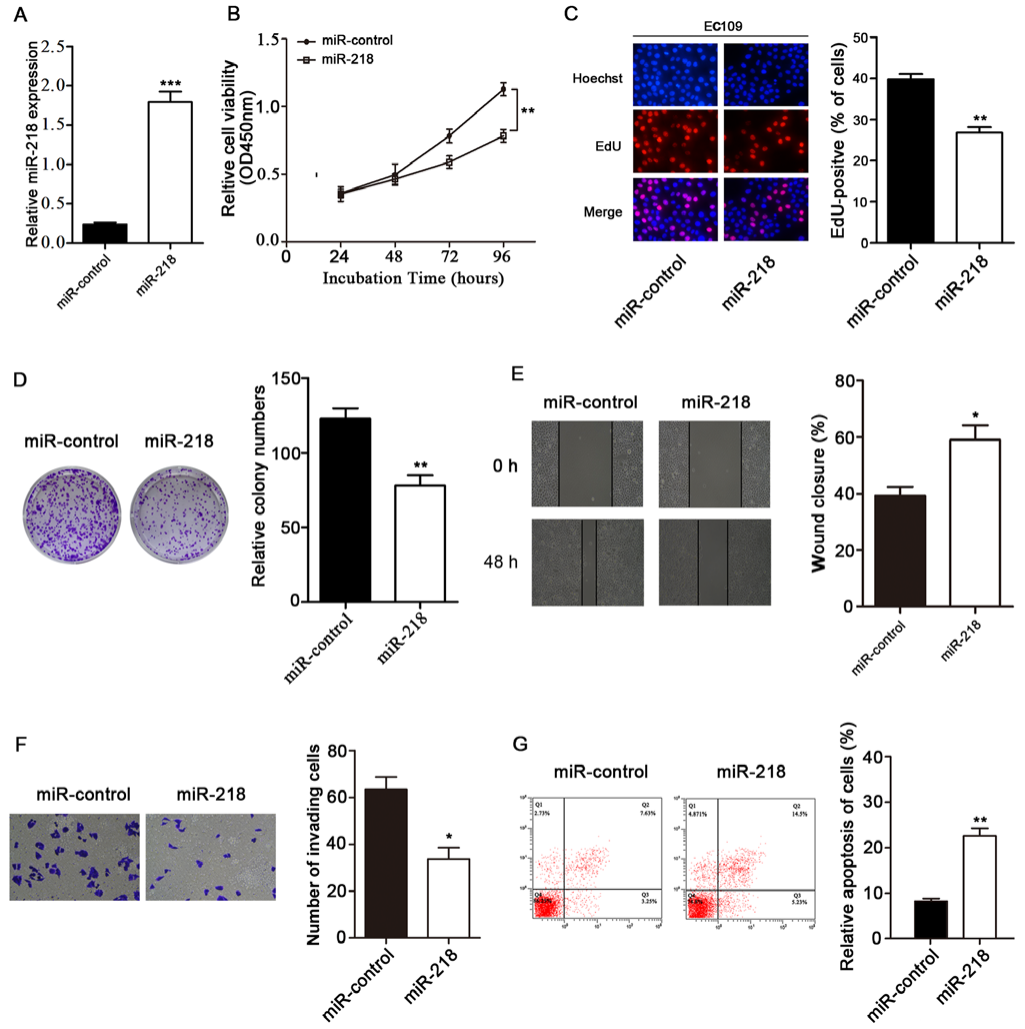
Inhibition of EC109 cell proliferation, migration and invasion by miR-218 and the increase in EC109 cell apoptosis induced by miR-218. (A) Expression of miR-218 measured by RT-qPCR in EC109 cells transfected with miR-218 mimics or miR-control. (B) MTT assay of relative EC109 cell viability at different time points (24, 48, 72 and 96 h) after transfection with miR-218 mimics or miR-control. (C) Effect of miR-218 on EC109 cell proliferation determined by EdU staining. Blue, Hoechst 33342 labeling of cell nuclei; red, EdU labeling of nuclei of proliferative cells. Quantitative data showed the percentage of EdU-positive cells (number of red vs. blue nuclei). (D) Effect of miR-218 overexpression on colony formation of EC109 cells. (E) Effect of miR-218 on migration of EC109 cells by wound healing assay. (F) Transwell invasion assay of EC109 cells treated with miR-218 mimics. (G) Effect of miR-218 on EC109 cell apoptosis determined by flow cytometry. Q2 + Q3 represent the total apoptotic rate. Data are presented as the means ± SD from 3 replicate samples. ^*^P<0.05 and ^**^P<0.01 vs. control.

Since the decreased expression of miR-218 was detected in the ESCC tissues and cell lines, we hypothesized that miR-218 acts as tumor suppressor gene and regulates tumor formation and development. We then investigated whether treatment with miR-218 exerts an inhibitory effect on the growth of ESCC cells. We found that miR-218 expression in the mimics-transfected group was significantly higher than that in the miR-control-transfected group (Fig. 2A). The corresponding cell growth curves indicated that the OD450 of the miR-218 group was significantly decreased on the fourth day post-transfection in the EC109 cells (Fig. 2B). In addition, we found that the extent of EdU staining was decreased in the EC109 cells transfected with the miR-218 mimics. EdU assay revealed that the number of EC109 cells was significantly decreased in the group transfected with miR-218 mimics (Fig. 2C). Moreover, colony formation assay also validated that transfection with miR-218 mimics was effective. The restoration of miR-218 expression markedly inhibited clonogenic cell growth, since transfection with miR-218 mimics led to the inhibition of colony formation compared to the miR-control-transfected group (P<0.01; Fig. 2D). These results indicate that miR-218 has the ability to effectively suppress ESCC cell growth.

In addition, the effects of miR-218 on cell migration and invasion were detected in the EC109 cells. Our results revealed that the overexpression of miR-218 significantly suppressed cell migration and invasion in the ESCC cells (P<0.05; Fig. 2E and F).

Next, we examined the effects of miR-218 restoration on the induction of apoptosis in EC109 cells. The EC109 cells were transfected with the miR-218 mimics for 72 h. The apoptosis of the miR-218-transfected cells was significantly increased compared with that of the miR-control-transfected cells (P<0.01; Fig. 2G).

### miR-218 downregulates BMI1 expression by targeting the 3′-UTR of BMI1 mRNA

**Figure 3:**
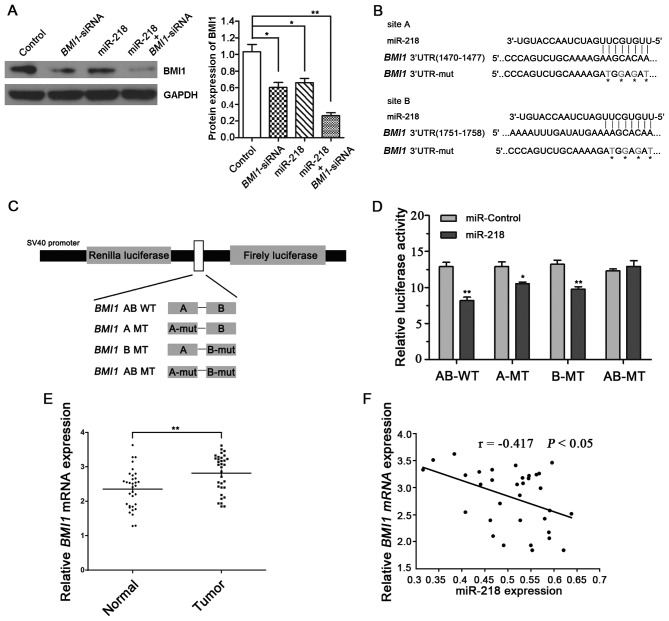
Identification of *BMI1* as target of miR-218 in EC109 cells and ESCC tissues. (A) Detection of BMI1 expression by western blot analysis following transfection of EC109 cells with miR-218 mimics, *BMI1*-siRNA, or co-transfection of EC109 cells with miR-218 mimics and *BMI1*-siRNA. (B) Prediction of the potential binding sites of miR-218 in the 3′-UTR of *BMI1* using bioinformatics software. (C) Schematic diagram showing cloning strategy of the predicted miR-218 binding sites of *BMI1*-3′-UTR into psiCHECK-2 luciferase vector. Predicted duplex formation between miR-218 and the wild-type/mutant of miR-218 binding sites was indicated. (D) Luciferase activity of the wild-type or mutant *BMI1*-3′-UTR reporter gene in EC109 cells transfected with control oligo or miR-218 mimics. AB-WT represents wild-type of both site A and site B; A-MT and B-MT represent single mutant type of site A or site B, respectively. AB-MT represents double mutant type of both site A and site B. Differences in luciferase activity were assessed by a two-tailed t-test. Data are presented as the means ± SD from 3 replicate samples. A scrambled sequence was used as a control. Relative *Renilla* luciferase activity was obtained after normalizing to Firefly luciferase activity. (E) Average mRNA expression level of *BMI1* in ESCC tissue samples and adjacent normal tissue samples. ^*^P<0.05 and ^**^P<0.01. (F) Inverse correlation between miR-218 and *BMI1* mRNA levels in the 33 ESCC tissue samples shown by Spearman’s correlation analysis.

Our results thus far suggested that the ectopic expression of miR-218 exerted an effect on ESCC cell proliferation, invasion and apoptosis. The upregulation of miR-218 expression has a potentially tumor suppressive funciton for EC development. To determine whether miR-218 plays a negative regulatory role in downregulating proteins and genes, we used TargetScan, miRDB and miRecords software to predict the potential genes inolved. Based on the results of TargetScan, we found 1,067 conserved sites and 279 poorly conserved sites among biological species. Among these genes, we found that *BMI1* was previously identifed in glioma stem cells by directly targeting the 3′-UTR of *BMI1* (15). Therefore, we selected *BMI1* as a determinant factor for *BMI1* expression in ESCC cells based on the prediction by a variety of software and as potential target gene of miR-218. We first investigated the protein expression of BMI1 in ESCC cells by western blot analysis, and found that the protein expression of BMI1 was decreased by the restoration of miR-218 expression (Fig. 3A). BMI1 protein expression was also downregulated by transfection with *BMI1*-siRNA.

The potential binding sites of miR-218 in the 3′-UTR of *BMI1* were perdicted using 2 types of bioinformatics software (TargetScan and miDRB). We found there were 2 binding sites, and one of them was a poorly conserved sequence. In order to confirm that miR-218 regulates the expression of *BMI1* by directly binding to the putative binding sites in its 3′-UTR, we constructed a luciferase reporter plasmid containing the full-length wild-type *BMI1* 3′-UTR and mutant-type *BMI1* 3′-UTR that had 4 point mutations predicted to disrupt miR-218 binding in each of the seed match regions (Fig. 3B and C). Co-transfection of the EC109 cells was performed with miR-218 mimics or miR-control. The luciferase reporter activities of both wild-type *BMI1* 3′-UTR binding sites were significantly decreased following transfection with miR-218 mimics compared with the miR-control; however, no significant difference was observed in the 2 point mutation groups (BMI1-AB-MT) (Fig. 3D). In conclusion, our results revealed that miR-218 directly binds to the 3′-UTR of *BMI1* mRNA and downregulates its expression.

We further investigated the *BMI1* mRNA expression levels in 33 pairs of ESCC samples by RT-qPCR. We found that the mRNA expression of *BMI1* was significantly higher in the cancer specimens compared with their paired non-tumor specimens (n=33; P<0.01; Fig. 3E). miR-218 expression and the mRNA levels of *BMI1* showed a significant inverse correlation in the 33 ESCC specimens by Spearman’s correlation analysis (r=−0.417; P<0.05; Fig. 3F). These data further suggest that the downregulation of miR-218 inversely correlates with the upregulation of *BMI1* in ESCC tissues.

### Knockdown of BMI1 by siRNA shows the same phenocopy as the effect of miR-218 on ESCC cells

**Figure 4:**
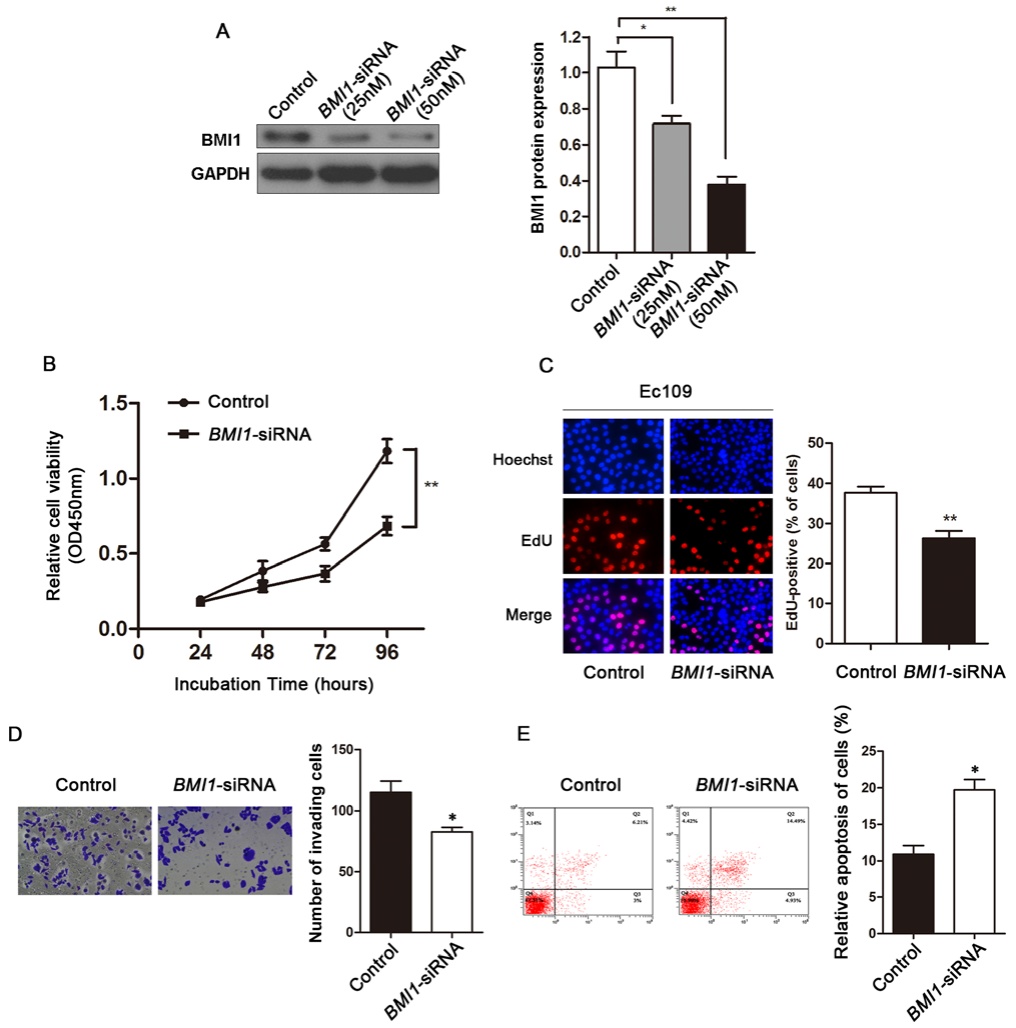
Effects of the downregulation of *BMI1* expression on the phenotype of EC109 cells. (A) Determination of BMI1 protein levels by western blot analysis. EC109 cells were transfected with 25 nM *BMI1*-siRNA, 50 nM *BMI1*-siRNA or control oligo. GAPDH was used as a loading control. The relative values of *BMI1* expression in the cells were calculated following normalization to GAPDH using ImageJ software. (B) MTT assay of EC109 cell growth at different time points (24, 48, 72 and 96 h) following transfection with *BMI1*-siRNA or control oligo. (C) Effect of *BMI1* on EC109 cell proliferation determined by EdU staining. (D) Transwell invasion assay of EC109 cells transfected with *BMI1*-siRNA. (E) Effect of *BMI1* on EC109 cell apoptosis determined by flow cytometry. Data are presented as the means ± SD from 3 replicate samples. ^*^P<0.05 and ^**^P<0.01 vs. control.

To explore the effects of *BMI1* on ESCC, we used *BMI1*-siRNA to knockdown *BMI1* expression in the EC109 cells. The protein expression level of BMI1 was significantly reduced in the *BMI1*-siRNA-transfected EC109 cells compared with the siRNA-control-transfected EC109 cells (Fig. 4A). MTT assay revealed that the growth rate of EC109 cells transfected with *BMI1*-siRNA was significantly lower than that of the siRNA-control-transfected cells (Fig. 4B). In order to further elucidate the effects of *BMI1*-siRNA on cancer cell proliferation, we used EdU incorporation assay to determine the effects of *BMI1* on ESCC cell proliferation. EdU assay revealed that cell number was significantly reduced in the *BMI1*-siRNA-transfected EC109 cells (Fig. 4C). Transwell assay revealed that *BMI1* knockdown inhibited the invasion of ESCC cells (Fig. 4D). The number of apoptotic cells were significantly increased in the *BMI1*-siRNA-transfected EC109 cells compared with the siRNA-control-transfected cells (Fig. 4E).

### Overexpression of BMI1 reverses the effects of miR-218 on ESCC cells

**Figure 5:**
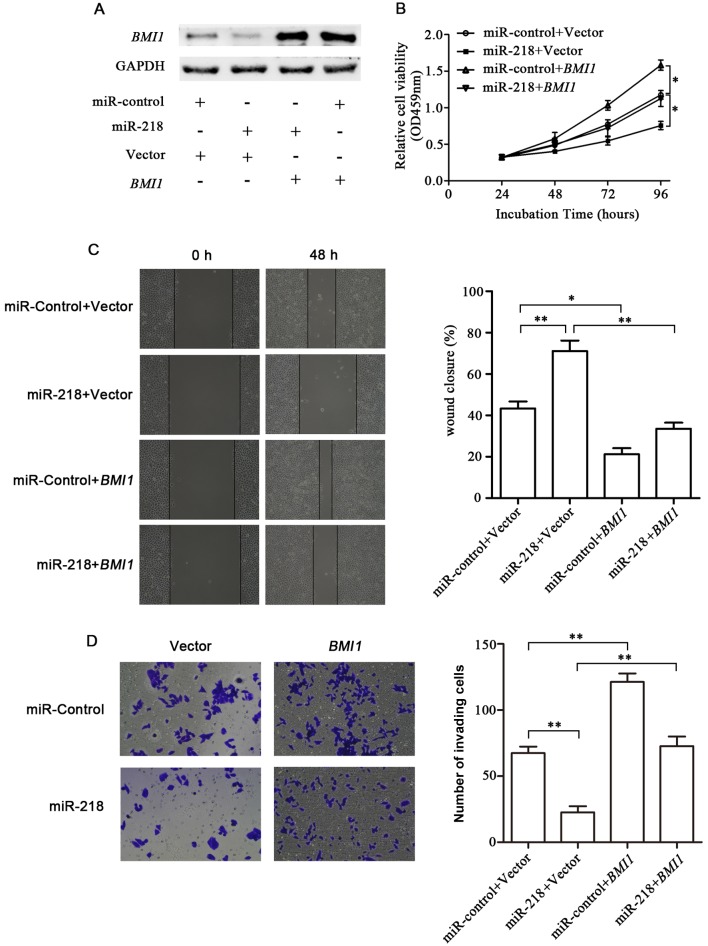
Partial reversal of the inhibitory effects of miR-218 on the ESCC cell phenotype by *BMI1*. (A) Western blot analysis of BMI1 protein levels in EC109 cells which were co-transfected with miR-218 mimics or miR-control and the *BMI1* plasmid (without 3′-UTR) or vector control. (B) MTT assay of relative EC109 cell viability. (C) Wound healing assay. (D) Transwell invasion assay. Data are presented as the means ± SD from 3 replicate samples. ^*^P<0.05 and ^**^P<0.01.

Since our results suggested that miR-218 inhibited cell growth and invasion through teh downregulation of *BMI1*, we explored whether *BMI1* is a direct functional mediator of the inhibitory effects of miR-218. The EC109 cells were transfected with *BMI1* plasmid without the 3′-UTR or control plasmid, in combination with transfection with miR-218 or miR-control. Forty hours after the *BMI1* plasmid or the control vector were co-transfected with the miR-218 or miR-control into the cells, western blot analysis confirmed that miR-218 downregulated *BMI1* expression; however, this effect was reversed by transfection with *BMI1* plasmid (Fig. 5A). It was also suggested that the *BMI1* construct without the 3′-UTR was insensitive to miR-218-mediated inhibition. MTT assay revealed that the overexpression of *BMI1* significantly reversed miR-218-induced cell growth inhibition (Fig. 5B). Furthermore, *BMI1* partially reversed the inhibitory effects of miR-218 on EC109 cell migration and invasion (Fig. 5C and D). These results suggested that the overexpression of *BMI1* reversed the effects of the aberrant expression of miR-218 on the phenotype of ESCC cells.

## Discussion

In the present study, we demonstrated that miR-218 expression was significantly downregulated in the ESCC tissues compared with the adjacent normal tissue. This result was consistent with that of a previous study (16). We also found that the decreased expression of miR-218 was associated with the ESCC differentiation stage and the metastasis of ESCC cells, as well as with the clinicopathological stages of ESCC (miR-218 expression was lower in the samples of stage III ESCC than in the samples of stage I and II ESCC). Moreover, we demonstrated that miR-218 negatively regulated *BMI1* expression in ESCC cells, suggesting an important role for miR-218 dysregulation in tumorigenesis and the metastasis of ESCC cells.

miRNAs are a class of endogenous small non-coding RNA molecules which function at the post-transcriptional level by partially combination with the 3′-UTR of the target mRNA (17). Ample evidence indicates that miRNAs play pivotal roles in regulating cell differentiation, proliferation and apoptosis (18). A number of miRNAs have been reported to regulate ESCC development. Previous studies have demonstrated that miR-218 mainly functions as tumor suppressor in various of types of cancer, including glioma, nasopharyngeal, gastric and colon cancers (15,19–21), but no consensus has yet been reached on the relevance of miR-218 in the development and progression of ESCC. In the present study, we demonstrated that miR-218 was significantly downregulated in ESCC tissues.

We then investigated whether the dysregulation of miR-218 is responsible for ESCC cell growth. First, we transfected EC109 cells with miR-218 mimics and analyzed cell growth *in vitro*, and found that cell growth was suppressed by miR-218. We also found that the overexpression of miR-218 decreased the migratory and invasive potential of the ESCC cells. Moreover, the apoptotic rate of the EC109 cells increased following transfection with miR-218 mimics. These findings suggest a important role of miR-218 ESCC; the downregulation of in miR-218 in ESCC cells promotes anchorage-independent cell growth, resistance to apoptosis and is a prerequisite for metastasis.

*BMI1* is a member of the polycomb group (PcG) of transcription repressors that repress targeted gene transcription through an epigenetic mechanism (22). In addition to its effect on EC, *BMI1* is also a pivotal factor in many other types of cancer. It has been found that *BMI1* is highly expressed in cervical, breast and ovarian tumor tissue compared to normal tissue (23,24). Immunohistochemical analysis has shown that *BMI1* expression in glioma tissues is markedly higher compared with ther normal tissue (25). The aberrant expression of *BMI1* is involved in cell proliferation, epithelial-mesenchymal transition (EMT), tumor invasion and metastasis in many types of cancer (26–29). Our results revealed that the downregulation of *BMI1* inhibited ESCC cell proliferation. In agreement with our results, the increased expression level of *BMI1* has been shown to promote the proliferation of ovarian cancer cells (30). *BMI1* has been shown to be a target of other miRNAs, including miR-128, miR-15a and miR-203 and these miRNAs regulate cancer cell proliferation, differentiation and invasion (31–33).

Our study demonstrated that *BMI1* was directly regulated by miR-218 in ESCC. It contains two binding sites, one is a conserved site, the other is a poorly conserved site. Using a luciferase reporter assay, we demonstrated that miR-218 directly targeted *BMI1* by binding to the 2 potential 3′UTR binding sites. We also identified miR-218 as a potential tumor suppressor that regulated cell proliferation and promoted apoptosis in ESCC cells. In agreement with our results, miR-218 has been shown to have a similar function in other types of cancer (34–36), thus, confirming the tumor suppressor role of miR-218. Concomitantly, similar results were obtained by the downregulation of *BMI1* using siRNA which better explained that miR-218 functions by inhibiting *BMI1* expression. Furthermore, the inverse correlation between miR-218 and *BMI1* mRNA expression was significant in the ESCC tissues. This result indicates that the downregulation of miR-218 is a remarkable event that may correlate with the upregulation of *BMI1* expression. Therefore, the decrease in miR-218 expression contributes to an enhanced cell proliferation and metastasis observed in ESCC by influencing *BMI1* expression.

In conclusion, in the present study, miR-218 was found to be significantly decreased expression in ESCC, and miR-218 targets *BMI1* and downregulates its expression in ESCC cells, which is important in regulating cancer cell growth and metastasis. Our data suggest that miR-218 and *BMI1* may function as novel biomarkers for monitoring the initiation and development of ESCC.

## Figures and Tables

**Table I:** Primers used for reverse transcription or amplification of the mature miR-218, U6 and *BMI1* mRNA.

Name	Sequence, 5′→3′
RT primers	
U6	CGAGCACAGAATCGCTTCACGAATTTGCGTGTCAT
miR-218	GTCGTATCCAGTGCAGGGTCCGAGGTATTCGCACTGGATACGACACATGGTTA
qRT-PCR primers	
U6	F: CGAGCACAGAATCGCTTCA; R: CTCGCTTCGGCAGCACATAT
GAPDH	F: GAAGGTGAAGGTCGGAGTC; R: GAAGATGGTGATGGGATTTC
*BMI1*	F: GTGCTTTGTGGAGGGTACTTCAT; R: TTGGACATCACAAATAGGACAATACTT

F, forward; R, reverse.

**Table II:** Correlation between the miR-218 levels and clinicopathological characteristics.

Clinicopathological variables	N	miR-218 expression	P-value
Gender			
Male	25	0.511±0.133	0.561
Female	8	0.549±0.141	
Age (years)			
≤60	19	0.525±0.118	0.326
>60	14	0.584±0.145	
Tumor location			
Up/middle	20	0.538±0.131	0.217
Lower	13	0.570±0.114	
Lymph node metastasis			
Negative	24	0.545±0.073	<0.05
Positive	9	0.442±0.142	
Differentiation			
Well	13	0.614±0.121	<0.01
Moderate/poor	20	0.422±0.117	
TNM stage			
I/II	26	0.621±0.185	<0.01
III	7	0.475±0.048	

## References

[1] Jemal A, Bray F, Center MM, Ferlay J, Ward E, Forman D (2011). Global cancer statistics. CA Cancer J Clin.

[2] Jemal A, Center MM, DeSantis C, Ward EM (2010). Global patterns of cancer incidence and mortality rates and trends. Cancer Epidemiol Biomarkers Prev.

[3] Song Q, Liu H, Wang J (2014). Dinner-to-bed time and post-dinner walk: new potential independent factors in esophageal cancer development. J Cancer Res Clin Oncol.

[4] Wong TS, Man OY, Tsang CM (2011). MicroRNA let-7 suppresses nasopharyngeal carcinoma cells proliferation through downregulating c-Myc expression. J Cancer Res Clin Oncol.

[5] Wang C, Wang X, Liang H (2013). miR-203 inhibits cell proliferation and migration of lung cancer cells by targeting PKCalpha. PLoS One.

[6] Thulin P, Wei T, Werngren O (2013). MicroRNA-9 regulates the expression of peroxisome proliferator-activated receptor delta in human monocytes during the inflammatory response. Int J Mol Med.

[7] Ratner ES, Tuck D, Richter C (2010). MicroRNA signatures differentiate uterine cancer tumor subtypes. Gynecol Oncol.

[8] Zhao BS, Liu SG, Wang TY (2013). Screening of microRNA in patients with esophageal cancer at same tumor node metastasis stage with different prognoses. Asian Pac J Cancer Prev.

[9] Kinoshita T, Hanazawa T, Nohata N (2012). Tumor suppressive microRNA-218 inhibits cancer cell migration and invasion through targeting laminin-332 in head and neck squamous cell carcinoma. Oncotarget.

[10] Yamamoto N, Kinoshita T, Nohata N (2013). Tumor suppressive microRNA-218 inhibits cancer cell migration and invasion by targeting focal adhesion pathways in cervical squamous cell carcinoma. Int J Oncol.

[11] Tatarano S, Chiyomaru T, Kawakami K (2011). miR-218 on the genomic loss region of chromosome 4p15.31 functions as a tumor suppressor in bladder cancer. Int J Oncol.

[12] Uesugi A, Kozaki K, Tsuruta T (2011). The tumor suppressive microRNA miR-218 targets the mTOR component Rictor and inhibits AKT phosphorylation in oral cancer. Cancer Res.

[13] Jacobs JJ, Kieboom K, Marino S, DePinho RA, van Lohuizen M (1999). The oncogene and Polycomb-group gene bmi-1 regulates cell proliferation and senescence through the ink4a locus. Nature.

[14] Zheng F, Liao YJ, Cai MY (2012). The putative tumour suppressor microRNA-124 modulates hepatocellular carcinoma cell aggressiveness by repressing ROCK2 and EZH2. Gut.

[15] Tu Y, Gao X, Li G (2013). MicroRNA-218 inhibits glioma invasion, migration, proliferation, and cancer stem-like cell self-renewal by targeting the polycomb group gene Bmi1. Cancer Res.

[16] Chen Z, Li J, Tian L (2014). MiRNA expression profile reveals a prognostic signature for esophageal squamous cell carcinoma. Cancer Lett.

[17] Huang X, Lv W, Zhang JH, Lu DL (2014). miR96 functions as a tumor suppressor gene by targeting NUAK1 in pancreatic cancer. Int J Mol Med.

[18] Wang A, Landen NX, Meisgen F (2014). MicroRNA-31 is overexpressed in cutaneous squamous cell carcinoma and regulates cell motility and colony formation ability of tumor cells. PloS One.

[19] Alajez NM, Lenarduzzi M, Ito E (2011). MiR-218 suppresses nasopharyngeal cancer progression through downregulation of survivin and the SLIT2-ROBO1 pathway. Cancer Res.

[20] Tie J, Pan Y, Zhao L (2010). MiR-218 inhibits invasion and metastasis of gastric cancer by targeting the Robo1 receptor. PLoS Genet.

[21] He X, Dong Y, Wu CW (2012). MicroRNA-218 inhibits cell cycle progression and promotes apoptosis in colon cancer by downregulating BMI1 polycomb ring finger oncogene. Mol Med.

[22] van der Lugt NM, Domen J, Linders K (1994). Posterior transformation, neurological abnormalities, and severe hematopoietic defects in mice with a targeted deletion of the bmi-1 proto-oncogene. Genes Dev.

[23] Vrzalikova K, Skarda J, Ehrmann J (2008). Prognostic value of Bmi-1 oncoprotein expression in NSCLC patients: a tissue microarray study. J Cancer Res Clin Oncol.

[24] Gavrilescu MM, Todosi AM, Anitei MG, Filip B, Scripcariu V (2012). Expression of bmi-1 protein in cervical, breast and ovarian cancer. Rev Med Chir Soc Med Nat Iasi.

[25] Wu Z, Wang Q, Wang L (2013). Combined aberrant expression of Bmi1 and EZH2 is predictive of poor prognosis in glioma patients. J Neurol Sci.

[26] Hamada S, Satoh K, Masamune A, Shimosegawa T (2012). Regulators of epithelial mesenchymal transition in pancreatic cancer. Front Physiol.

[27] Paranjape AN, Balaji SA, Mandal T, Krushik EV, Nagaraj P, Mukherjee G, Rangarajan A (2014). Bmi1 regulates self-renewal and epithelial to mesenchymal transition in breast cancer cells through Nanog. BMC Cancer.

[28] He Q, Liu Z, Zhao T, Zhao L, Zhou X, Wang A (2015). Bmi1 drives stem-like properties and is associated with migration, invasion, and poor prognosis in tongue squamous cell carcinoma. Int J Biol Sci.

[29] Chen Y, Lian G, Zhang Q, Zeng L, Qian C, Chen S, Huang K (2013). Overexpression of Bmi-1 induces the malignant transformation of gastric epithelial cells in vitro. Oncol Res.

[30] Xin T, Zhang FB, Sui GJ, Jin XM (2012). Bmi-1 siRNA inhibited ovarian cancer cell line growth and decreased telomerase activity. Br J Biomed Sci.

[31] Peruzzi P, Bronisz A, Nowicki MO (2013). MicroRNA-128 coordinately targets Polycomb Repressor Complexes in glioma stem cells. Neuro Oncol.

[32] Guo S, Xu X, Tang Y (2014). miR-15a inhibits cell proliferation and epithelial to mesenchymal transition in pancreatic ductal adenocarcinoma by down-regulating Bmi-1 expression. Cancer Lett.

[33] Okumura T, Shimada Y, Moriyama M (2014). MicroRNA-203 inhibits the progression of esophageal squamous cell carcinoma with restored epithelial tissue architecture in vivo. Int J Oncol.

[34] Kogo R, How C, Chaudary N, Bruce J, Shi W, Hill RP, Zahedi P, Yip KW, Liu FF (2015). The microRNA-218~Survivin axis regulates migration, invasion, and lymph node metastasis in cervical cancer. Oncotarget.

[35] Sui C, Xu F, Shen W, Geng L, Xie F, Dai B, Lu J, Zhang M, Yang J (2015). Overexpression of miR-218 inhibits hepatocellular carcinoma cell growth through RET. Tumour Biol.

[36] Nishikawa R, Goto Y, Sakamoto S, Chiyomaru T, Enokida H, Kojima S, Kinoshita T, Yamamoto N, Nakagawa M, Naya Y (2014). Tumor-suppressive microRNA-218 inhibits cancer cell migration and invasion via targeting of LASP1 in prostate cancer. Cancer Sci.

